# Implications of the COVID-19 pandemic on the shanghai, New York, and Pakistan stock exchanges

**DOI:** 10.1016/j.heliyon.2023.e17525

**Published:** 2023-06-26

**Authors:** Muhammad Aamir, Nazeem Khan, Muhammad Naeem, Muhammad Bilal, Faisal Khan, Saleem Abdullah

**Affiliations:** a Department of Statistics, Abdul Wali Khan University Mardan, Pakistan; b Department of Mathematical Sciences, Balochistan University of Information Technology, Engineering and Management Sciences (BUITEMS), Quetta, Pakistan; c Department of Electrical and Electronic Engineering, College of Science and Engineering, National University of Ireland Galway, Ireland; d Department of Mathematics, Abdul Wali Khan University Mardan, Pakistan

**Keywords:** Auto-regressive distributed lag (ARDL), COVID-19, New York stock exchanges, Pakistan, And shanghai

## Abstract

This research aims to determine the impact of COVID-19 on the stock markets of Pakistan (Islamabad), China (Shanghai), and the United States of America (New York). These three stock markets were chosen to demonstrate the variation in the degree of influence based on varied times in which the respective nations were impacted by COVID-19. COVID-19, a pandemic virus, was still present in China in December 2020. The one-year timeline helps us understand the pattern of the effect on different stock markets that show onward to guide us to indicate that in this situation, the lack of economic movement (due to the lockdown) had a more negative effect on stock prices than the increase in the number of new confirmed cases of the COVID-19 virus. This study was carried out to assess the influence of COVID-19 on the financial sectors, including the stock market. The effects were assessed by employing the Autoregressive Distributed Lag Model (ARDL) to demonstrate correlations between three stock markets (Pakistan, Shanghai, and New York) and COVID-19 instances. The study's major goal is to demonstrate the differences in the three countries' levels of influence. We got empirical results and discovered that the confirmed cases had a detrimental influence on three stock exchanges. However, all three countries saw an increase in the number of recovery cases. The number of deaths was minor for Pakistan and China but had a detrimental impact on the New York Stock Exchange.

## 1 Introduction

Numerous sicknesses or diseases, pandemics, and epidemics have struck the world, causing not only economic disruption but also the emergence of various diseases as a result of pandemic clues, damage, psychological issue, disaster, unrest, and terror fear. Some annihilative pandemics and epidemics were still expected to erupt in China in December 2020, according to COVID-19. The viral propagation of this pandemic is frequently quicker than that of any prior pandemic. This pandemic has infected millions of people and is presently expanding rapidly. A pandemic poses a significant risk not just to a single nation, but to the entire world, because globalization connects the country of one individual, and the virus has a secondary impact. The pandemic had a financial impact on many economies throughout the world, producing suffering, misery, and high mortality rates. In December 2019, a new viral case was detected in Wuhan, China. The virus is a cold and severe acute respiratory syndrome (SARS) virus [1]. Coronavirus symptoms include shortness of breath or difficulty breathing, cold or fever, sore throat, fatigue, muscle or bodily aches, loss of smell or taste, headache, diarrhea, and nausea or vomiting. A patient died as a result of COVID-19 on January 11, 2020, and the therapy was unsuccessful [2]. During the outbreak, COVID-19 cases were reported in South Korea, Australia, France, Malaysia, Singapore, Taiwan, and Nepal. Thousands of people died and millions were sick by the end of April. Following that, South Korea was the second country to face a huge COVID-19 pandemic, with around 9 million positive COVID-19 cases reported globally, as well as 472, 539 fatalities [3]. The number of positive cases and recoveries has increased since the disease's inception. However, the number of cases in Europe has declined while it has increased in Asia and America. On March 11, 2020, WHO declared the outbreak to be worldwide, affecting more than 170 countries [4]. COVID-19 has pushed the world towards low-cost markets, followed by a catastrophic slump. Protective events, such as community separation and lockdowns, must illustrate their importance to a larger extent, but they come at a cost in the form of reduced commercial sales and smoothing the stability of diverse organizations. COVID-19-related financial expenditures have also had an influence on global stock markets. Confirmed pandemic instances on global stock markets have occurred in practically every place, and the Pakistani stock exchange is also one of the linkages impacted by COVID-19.

The Autoregressive Distributed Lag Model (ARDL) is an ordinary least square (OLS) based model that is appropriate for both stationary and non-stationary time series with mixed order of variables I(0), I(1) to estimate relationships between three stock exchanges (Pakistan, Shanghai, and New York) and COVID-19, which contains new confirm, death, and recover cases in due course, to investigate the impact of COVID-19 on the financial sectors, including the stock market. The goal of this study is to see how COVID-19 affects three stock markets. The pandemic has touched almost every business on the earth. As a consequence, the current study will help in evaluating the relationship between COVID-19 events and stock market exchanges, particularly for the three COVID-19-affected countries. The research perspective is that stock analysts use stock analysis to forecast the future activities of an instrument, industry, or market for investors. And the traders decide which stocks to purchase and sell. By studying and analyzing past and current market data, investors and traders may get a competitive advantage in the market.

### 1.1 Literature review

Several incidents were cited to indicate how the ongoing epidemic is harming the stock market. Forecasting COVID-19 is also important, and numerous ways have been developed by researchers. The researchers [5] developed and implemented the Kalman filter in the top four countries: the United States, India, Pakistan, and Russia. The Kalman filter is applied to both the smoothed data and the filter technique. The researchers also find the forecast for the top four countries for the following fifteen days. Confirmed cases grow in the United States and India while decreasing in Brazil and Russia; recover cases increase in the United States, India, and Brazil while decreasing in Russia; and deaths decrease in the United States while increasing in Russia, India, and Brazil. Similarly, the authors [6] devised an ensemble learning-based strategy and compared the results to the standard ARIMA model. Furthermore, based on their findings, ensemble learning outperforms the traditional ARIMA model. Furthermore, the author forecasted recovered instances for Pakistan in the next fifteen days. The influence of COVID-19 on the stock market and potential participation plans by studying various companies such as travel-related enterprises, technology, and gold performance [7]. The learning is systematic, and the pandemic might have opposing impacts in the long run and in the short run. COVID-19 has impacted the stock markets of 21 nations. It has been discovered that the epidemic had a detrimental impact on the affected country's stock market, causing a quick drop in stock prices and return. The researchers [8] utilized a simple regression model to determine the effect of COVID-19 on the Chinese and American economies and while [9] used regression model as an input machine learning approaches to predict the COVID cases. The study examined short-term data and discovered that COVID-19 had a considerable favorable influence on the New York Dow Jones index and the Shanghai stock market. Authors [10] analyzed the stock market during the epidemic. They employed algorithms to determine the impact of the epidemic on the US stock market. According to the research study, the major source of government limits on marketable activity in a service-oriented economy was voluntary social isolation. The COVID-19 virus shook global financial markets, resulting in an irregular or unexpected backdrop with significant fluidity points. Authors [11] investigated pollution and its impact on individuals. Sequential and geographical autocorrelations to investigate the dynamics of country-wide infection growth rates and discovered significant values [12]. The researchers [13] employed price gradient analysis to determine how the current pandemic affects housing worth. The authors [14] underlined that COVID-19 has an impact on registered insurance manufacturing enterprises.

The researchers [15] investigated the total number of 10th developing marketplaces and discovered that COVID-19 affected the most firms. COVID-19 to research emerging markets. COVID-19 variation has an impact on economic markets [16]. According to Ref. [17], public concern, together with constraints and isolation, has an impact on market companies. They [18] showed that gold and oil were useless during the pre-epidemic corona. Later, depositors might discover moneymaking methods based on market shortcomings to achieve uneven returns. On the contrary [19], proposed that ideal savings include sightseeing manufacturing, skill sector, industrial holidays, and gold. Authors [20] criticizes the firms' response to COVID-19 in numerous ways, because many segments were shielded during the separation stage, and it verified that the companies will be vulnerable to the epidemic. The energy sector had the most irregular negative profits of any category [21]. Chinese industrial section was hardly affected by COVID-19, although the other sectors, such as building construction, computer services and software, fitness maintenance, and community service, were less affected [22].

Economic markets resisted the flight-to-safety phenomena, which resulted in a clear failure in asset values and increased global instability. Authors [23] investigated and concluded that the casualty rate had a significant impact on economic instability, but the provenance of olive stocks was not as influenced. An economy is a government act, with subsequent responses reliant on experts [24], responds that a recurrence of possible illnesses is associated with a 4%–11% decrease in aggregate market value. COVID-19 instances are more than only death cases, indicates that COVID-19 affected the stock market [25]. The researchers [26] demonstrate that there are disparities in the number of death cases and new cases. Furthermore, people were severely affected by the coronavirus, but this had little effect on stock market yields outside of the United States, omitting the number of cases in China. The global expansion of COVID-19 resulted in a higher growth in earnings on independent safeties than in emerging and developing nations.

The authors [27] investigated the stock market impact of COVID-19 by studying several manufacturers such as transportation, manufacturing, metals, and technology, entertainment. In his investigation, the author discovered that the pandemic might have opposite effects in the short term and rises in the long run. The researchers [28] investigated COVID-19 and its effects on stock market conditions. They utilized the regression approach and discovered that the epidemic has a significant negative impact on the valuable country's stock market and can cause a direct fall in stock prices and returns. The authors [29] explored the commercial issues caused by COVID-19. In the first quarter of 2020, they used data from over 6,000 businesses from 56 countries. They were interested in learning how COVID-19 cases affected stock prices and corporate attributes. According to the study's findings, enterprises with superior pre-2020 finances had less experience with pandemics, fewer fixed managers, and larger social charge activities, and they were able to manage a little pandemic-induced stock price drop. Furthermore, the statistics reveal that corporations with larger corporate possession outperformed organizations with developed tenure of dodge assets. All the past literature helps in carrying out this study on COVID-19 pandemic impacts on the Shanghai, New York, and Pakistan Stock Exchanges.

### 1.2 General model formulation

The Autoregressive Distributed Lag (ARDL) model is a time-series data model in statistics and econometrics that illustrates a link between explanatory and dependent variables based on both current and lagged values of both explanatory and dependent variables. The general form of ARDL is as under in eq. (1):

(1) $${logy}_{t}{=\alpha }_{0}+\sum_{i=0}^{m}\beta i{logy}_{t-i}+\sum_{j=0}^{n}\delta j{logc}_{t-j}+\sum_{j=0}^{n}\gamma j{logd}_{t-j}+\sum_{j=0}^{n}\omega j{logr}_{t-j}+\varepsilon_{t}$$

Model (1) is a distributed lag model with finite lags, which is further simplified as follows in eq. (2).

(2) $$y_{t}{=\alpha }_{0}{+\beta }_{1}y_{t-1}{+\beta }_{2}y_{t-2}+\ldots ,\beta_{j}y_{t-j}{+\delta }_{1}c_{t}{+\delta }_{2}c_{t-1}\ldots ,\delta_{i}c_{t-j}+\gamma_{1}d_{t}+\gamma_{2}d_{t-1}+\ldots ,\gamma_{i}d_{t-j}+\omega_{1}r_{t}+\omega_{2}r_{t-1}+\ldots ,\omega_{i}r_{t-j}{+\varepsilon }_{t}$$

Where $y_{t}$ represents the number of daily stock exchanges in three separate countries at the time t: Pakistan, Shanghai, and New York while $\alpha_{0}$ represents the intercept term in the same course and $\beta_{1}y_{t-1}+\beta_{2}y_{t-2}+\ldots ,\beta_{j}y_{t-j}$ are the $q^{th}$ autoregressive lag order of the model. The regressors “confirm” “death” and recovery cases are denoted by $c_{t}$, $d_{t}$ and $r_{t}$ respectively. Whereas $\delta_{1}c_{t}+\delta_{2}c_{t-1}+\ldots ,\delta_{i}c_{t-j},\gamma_{1}d_{t}+\gamma_{2}d_{t-1}+\ldots ,\gamma_{i}d_{t-j}\;and\;\omega_{1}r_{t}+\omega_{2}r_{t-1}+\ldots ,\omega_{i}r_{t-j}$ are the lags in order of $c_{t}$, $d_{t}$ and $r_{t}$ respectively. The parameters β, δ, γ, and ω coefficients of the stock exchange, confirm, death and recovery while $\varepsilon_{t}$ denotes the error term.

The explicit form of the ARDL model is as follows in eq. (3):

(3) $$y_{t}=\alpha +\sum_{i=1}^{p}\pi iy_{t-1}+\sum_{j=0}^{r}\beta jx_{t-j}+\varepsilon_{t}$$

Where $y_{t}$ and $x_{t}$ of the values of regressand and regressor and $\varepsilon_{t}$ is the error term.

### 1.3 Research methodology

This section explains the research methodology and estimation strategy, which covers the unit root test and auto-regressive distributed lag (ARDL). The ARDL model may be extended to account for different stock exchange $y_{t}\text{,}$ confirm $c_{t}$, death $d_{t}$ and recover $r_{t}$.

Pakistan Stock Exchange (PSX) model is given as in eq. (4):

(4) $$y_{t}{\left(psx\right)=-\alpha }_{0}{-\beta }_{1}y_{t-1}{+\beta }_{2}y_{t-2}{-\beta }_{3}y_{t-3}{-\beta }_{4}y_{t-4}{-\delta }_{1}c_{t-1}{-\delta }_{2}c_{t-2}{+\omega }_{1}r_{t-1}{+\varepsilon }_{t}$$

Shanghai Stock exchange (SSE) model is given as in eq. (5):

(5) $$y_{t}{\left(sse\right)=-\alpha }_{0}{-\beta }_{1}y_{t-1}{-\beta }_{2}y_{t-2}{-\beta }_{3}y_{t-3}{-\beta }_{4}y_{t-4}{-\delta }_{t}c_{t}{+\delta }_{1}c_{t-1}{+\delta }_{2}c_{t-2}{+\omega }_{3}r_{t-3}{+\varepsilon }_{t}$$

New York Stock Exchange (NYSE) model is given as in eq. (6):

$$y_{t}\left(nyse\right)={-\alpha }_{0}{-\beta }_{1}y_{t-1}{-\beta }_{2}y_{t-2}{-\beta }_{3}y_{t-3}{-\beta }_{4}y_{t-4}{-\beta }_{5}y_{t-5}{+\delta }_{2}c_{t-2}{+\delta }_{3}c_{t-3}{+\delta }_{4}c_{t-4}$$

(6) $${+\delta }_{5}c_{t-5}{+\gamma }_{4}d_{t-4}{+\omega }_{4}r_{t-4}{+\varepsilon }_{t}$$

The negative coefficients of the variables indicated that there is a negative association between stock exchanges and the number of COVID-19 instances in eqs. (4), (5), (6).

### 1.4 Unit root test

Each variable in the model was tested for stationarity and non-stationarity using the Augmented Dickey-Fuller test and the Kwiatkowski-Phillips-Schmidt-Shin (KPSS) test and the results are presented in Table 1, Table 2.

**Table 1:** Summary statistics of PSX and COVID-19 cases.

Variables	Mean	Median	Minimum	Maximum	Std. Dev	Obs
PSX	38891	40095	27229	46934	4839	366
Confirm	1591	1236	0	8687	1405.81	366
Death	34.17	27	0	153	30.77	366
Recovery	1473	859	0	16813	2021.59	366

**Table 2:** ADF and KPSS test for Pakistan Stock Exchange (PSX).

	ADF Test			KPSS Test		
Variables	ADF statistics	P-Value	Decision	KPSS statistics	P-Value	Decision
PSX	−12.47	0.01	Stationary	0.01015	0.1	Stationary
Confirm	−15.91	0.01	Stationary	0.00917	0.1	Stationary
Death	−14.694	0.01	Stationary	0.00961	0.1	Stationary
Recovery	−15.986	0.01	Stationary	0.00961	0.1	Stationary

Table 1 shows five number summary statistics, while Table 2 shows the Kwiatkowski-Phillips-Schmidt-Shin and Augmented Dickey-Fuller unit results. Because all of the variables are stationary, the model (4) result is shown in Table 3.

**Table 3:** ARDL model of Pakistan stock exchange.

Coefficients	Estimate	Std. Error	t-value	p-value
Intercept	−7.865e-05	7.334e-04	−0.107	0.9147
LOG (Ct.t)	- 6.930e-03	3.269e-03	−2.120	0.0348 *
LOG (Ct.1)	−1.079e-02	5.184e-03	−2.081	0.0382 *
LOG (Ct.2)	−1.324e-02	6.075e-03	−2.180	0.0301 *
LOG (Rt.1)	2.937e-03	1.844e-03	1.592	0.0112 *
LOG (Yt.1)	−1.240e+00	5.367e-02	−23.108	<2e-16 ***
LOG (Yt.2)	−1.109e+00	7.745e-02	−14.314	<2e-16 ***
LOG (Yt.3)	−8.154e-01	7.661e-02	−10.644	<2e-16 ***
LOG (Yt.4)	−3.786e-01	5.290e-02	−7.158	6.36e-12 ***
Residual standard error: 0.01319	Multiple R-squared 0.6871		Adjusted R-squared: 0.662	
F-statistic: 27.36			p-value <2.2e-6	
Source: Author’s computation using R (dLagM) package				
*** represent 1%, **5% and *10% level of significance respectively				

The coefficient is adversely connected to the confirming cases LOG (Ct) and its current, first, and second lag at the 10% level, while the first lag of the recovery LOG (Rt) is positively significant at the 10% level and the deaths cases are inconsequential. Furthermore, from the first to fourth lags, the coefficient of a stock exchange response variable LOG (Yt) is significant (negative) at the 1% level. The whole model is highly significant at the 1%, 5%, and 10% levels, with p-values less than 2.2e-16 and Adjusted R-squared values of 66.2% and 68.71%, respectively.

**Fig. 1:**
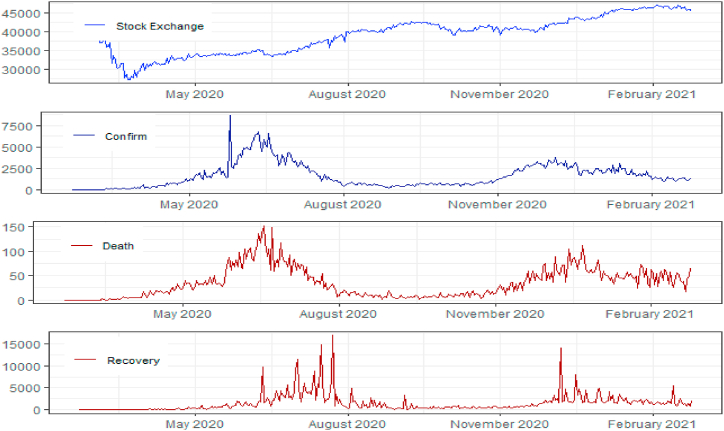
Pakistan stock exchange verses COVID-19 cases.

The fitted model is denoted by eq. (7) and represented graphically in Fig. 1:

$$y_{t}{\left(psx\right)=-0.0007-1.240y}_{t-1}{-1.10y}_{t-2}{-0.082y}_{t-3}{-0.037y}_{t-4}{-0.0069c}_{t}{-0.017c}_{t-1}$$

(7) $${-0.0133c}_{t-2}{+0.0029r}_{t-1}$$

Fig. 1 depicts the complete panel of the number of new pandemic i.e. confirmed, mortality, and recovery cases, which are compared to monthly movements in the Pakistan stock market. The figure demonstrates temporal similarities and differences in the trend line for each number of factors. The Pakistan stock market is plotted against the number of COVID-19 cases, having effect from February 2020 to February 2021. It is obvious that in the first month of March 2020 confirm cases began the Pakistan stock market went down with less record value on April 2, 6260 and confirm is 0 after April the trend line of the Pakistan stock market increased slowly fluctuating with the highest value of 46934 in February 2021 and 27229 at its lowest in March 2020 it fluctuated up to 72.3% in this period. We may deduce from the graph that when the number of pandemic cases increases or decreases after May 2020, the Pakistan stock market rises till the conclusion of the timeline period.

**Shanghai Stock Exchange** and COVID-19 implications summary statistics and corresponding Augmented Dickey-Fuller test and the Kwiatkowski-Phillips-Schmidt-Shin (KPSS) test results are presented in Table 4 and Table 5 respectively.

**Table 4:** Summary statistics of shanghai stock exchange and COVID-19 cases.

Variables	Mean	Median	Minimum	Maximum	Std. Dev	Obs
SSE	3144	3227	2660	3621	253.5	366
Confirm	83143	85800	547	98800	18006.55	366
Death	4139	4648	0	4803	1179.58	366
Recovery	228	19	0	3622	582.58	366

**Table 5:** ADF and KPSS tests for shanghai stock market.

	ADF Test			KPSS Test		
Variables	ADF statistics	P-Value	Decision	KPSS statistics	P-Value	Decision
SSE	−10.63	0.01	Stationary	0.0135	0.1	Stationary
Confirm	−6.89	0.01	Stationary	0.0083	0.1	Stationary
Death	−8.16	0.01	Stationary	0.0093	0.1	Stationary
Recovery	−11.43	0.01	Stationary	0.0103	0.1	Stationary

Table 4 shows five number summary statistics for SSE, while Table 5 shows the Kwiatkowski-Phillips-Schmidt-Shin and Augmented Dickey-Fuller unit results. Because all of the variables are stationary, the model (5) result is shown in Table 6.

**Table 6:** ARDL model of SSE & COVID-19 Cases.

Coefficients	Estimate	Std. Error	t-value	P-value
Intercept	−7.865e-05	7.334e-04	−0.107	0.9147
LOG (Ct.t)	−6.930e-03	3.269e-03	−2.120	0.0348 *
LOG (Ct.1)	−1.079e-02	5.184e-03	−2.081	0.0382 *
LOD (Ct.2)	−1.324e-02	6.075e-03	−2.180	0.0301 *
LOG (Rt.3)	4.084e-03	5.569e-03	0.733	0.0639 *
LOG (Yt.1)	−1.240e+00	5.367e-02	−23.108	<2e-16 ***
LOG (Yt.2)	−1.109e+00	7.745e-02	14.314	<2e-16 ***
LOG (Yt.3)	−8.154e-01	7.661e-02	−10.644	<2e-16 ***
LOG (Yt.4)	−3.786e-01	5.290e-02	−7.15	6.36e-12 ***
Residual standard error: 0.0131			Multiple R-squared: 0.6871	
Adjusted R-squared: 0.662	F-statistic: 27.36		p-value: <2.2e-16	
Source: Author’s computation using R (dLagM) package				
*** represent 1%, **5% and *10% level of significance respectively				

**Fig. 2:**
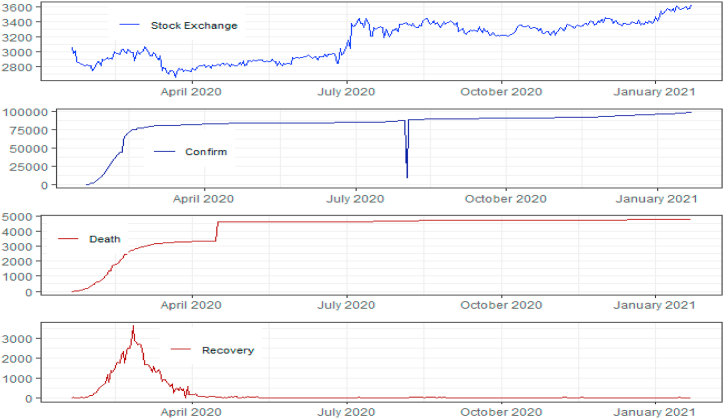
Shanghai stock exchange against COVID-19 cases.

The coefficients of the confirmed cases LOG (Ct) and its current, first, and second delays are all significant at the 10% level, but the third lag of the recovery LOG (Rt) is not. Furthermore, from the first to fourth lags, the coefficient of the China stock exchange response variable LOG (Yt) is significant (negative) at 1%. At the 1%, 5%, and 10% levels, the model is highly significant, with p-values less than 2.2e-16 and Adjusted R-squared values of 66.2% and 68.71%, respectively. The fitted model is denoted by eq. (8) and is explicitly illustrated in Fig. 2:

$$y_{t}{\left(sse\right)=-0.00007-1.240y}_{t-1}{-1.10y}_{t-2}{-0.082y}_{t-3}{-0,037y}_{t-4}\;{-0.0069c}_{t}$$

(8) $${-0.017c}_{t-1}{-0.0133c}_{t-2}{+0.0004r}_{t-3}$$

Fig. 2, in complete panel, portrays the pandemic's influence on the Shanghai stock exchange markets in terms of new confirmed, death, and recovery cases. The confirmed cases began to rise abruptly and considerably in January 2020 and continued to rise steadily until August 2020, when suddenly began to fall sharply, with the number of confirmed cases falling to 547, and then rising steadily until January 2021. Furthermore, we can see that in the early phases of the pandemic, the Shanghai stock market has a negative effect between February and April 2020, then steadily increases and decreases until the conclusion of the timeframe. The Shanghai stock exchange achieved a high of 3621 in January 2021 and a low of 2660 in March 2020. During this time, it fluctuated up to 36.12%. The figure shows that the Shanghai stock market had a negative impact in the early phases of recovery owing to new infected and death cases. After July 2020, the confirmed cases and April death cases stay stable and had no negative influence on the stock market.

**New York Stock Exchange (NYSE)** and COVID-19 implications summary statistics and corresponding Augmented Dickey-Fuller test and the Kwiatkowski-Phillips-Schmidt-Shin (KPSS) test results are presented in Table 7 and Table 8 respectively.

**Table 7:** Summary statistics of NYSE and COVID-19 cases.

Variable	Mean	Median	Minimum	Maximum	Std.Dev	Obs
NYSE	26988	27443	18592	31188	2627.6	366
Confirm	65729	39969	0	402270	71813.48	366
Death	1196	931	0	40313	2271.36	366
Recovery	64532.99	18425	0	501134	96869.9	366

**Table 8:** ADF and KPSS tests for NYSE.

	ADF Test			KPSS Test		
Variables	ADF statistics	P-Value	Decision	KPSS statistics	P-Value	Decision
NYSE	−14.76	0.01	Stationary	0.0930	0.1	Stationary
Confirm	−1.71	0.01	Stationary	0.0097	0.1	Stationary
Death	−14.69	0.01	Stationary	0.0084	0.1	Stationary
Recovery	−21.91	0.01	Stationary	0.0091	0.1	Stationary

Table 4 shows five number summary statistics for NYSE, while Table 5 shows the Kwiatkowski-Phillips-Schmidt-Shin and Augmented Dickey-Fuller unit results. Because all of the variables are stationary, model (6) result is shown in Table 9.

**Table 9:** ARDL model of NYSE & COVID-19 Cases.

Coefficients	Estimate	Std. Error	t value	P-value
(Intercept)	−0.0002981	0.0008780	−0.340	0.73444
LOG (Ct.2)	- 0.0245288	0.0100759	2.434	0.01556 *
LOG (Ct.3)	0.0324619	0.0099030	3.278	0.00118 **
LOG (Ct.4)	0.0296426	0.0091047	3.256	0.00127 **
LOG (Ct.5)	0.0135858	0.0069112	1.966	0.05033
LOG (Dt.2)	−0.0060942	0.0034588	2.434	0.07920.
LOG (Dt.4)	−0.0081194	0.0033701	−2.409	0.01664 *
LOG (Dt.5)	−0.0059702	0.0027781	−2.149	0.03251 *
LOG (Rt.4)	0.0015286	0.0023315	−0.656	0.01664 *
LOG (Yt.1)	−1.2221861	0.0571255	−21.395	<2e-16 ***
LOG (Yt.2)	−1.2651525	0.0838761	−15.084	<2e-16 ***
LOG (Yt.3)	−0.8877147	0.0937629	−9.468	<2e-16 ***
LOG (Yt.4)	−0.5102230	0.0815206	−6.259	1.49e-09 ***
LOG (Yt.5)	−0.1453392	0.0540557	−2.689	0.00761 **
Residual standard error: 0.01519		Adjusted R-squared: 0.6522		
Multiple R-squared: 0.6824	F-statistic: 22.64		p-value: <2.2e-16	
Source: Author’s computation using R (dLagM) package				
*** represent 1%, **5% and *10% level of significance respectively				

**Fig. 3:**
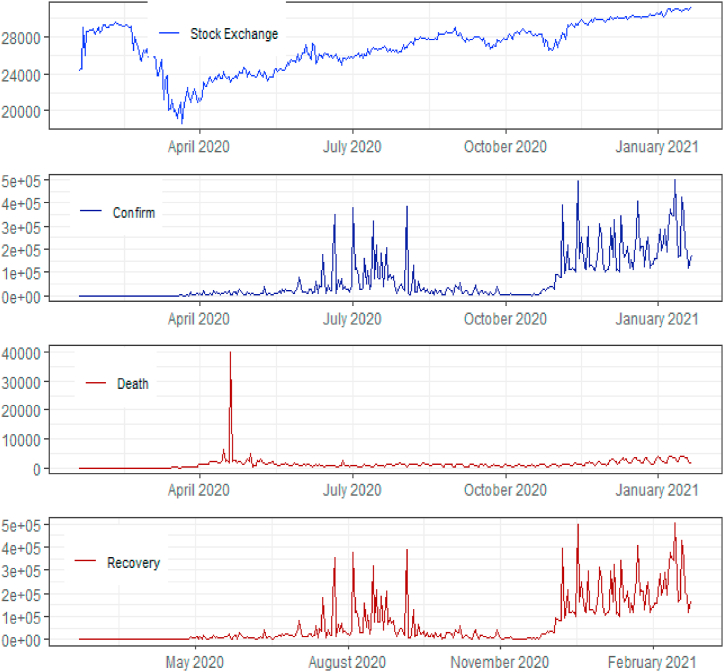
New York stock exchange against COVID-19 cases.

The coefficient favorably connected of confirm cases LOG (Ct) at the lags from third to fifth and its second lag is negatively significant at the level of 5%, third, and fourth lags are significant at the level of 1%, and lag fifth is significant at the level of 10% in Table 9. LOG (Dt) death is adversely significant at the 10% level at the second and fourth delays and the 5% level at the fifth lag. Furthermore, LOG (Rt) recovery is favorably significant at lag fourth at the level of 5%, whereas LOG (Yt) is significantly negative from lags first to fourth at the level of 0.1% and lag fifth at the level of 1%, with the adjusted R-squared 65.22% and multiple R-squared 68.24%. The fitted model is denoted by eq. (9) and represented graphically in Fig. 3:

$$\left(nyse\right)=-0.0029-1.2220y_{t-1}{-1.265y}_{t-2}{-0.888y}_{t-3}{-.0510y}_{t-4}{-0.145y}_{t-5}{-0.024c}_{t-2}$$

(9) $${+0.032c}_{t-3}{+0.029c}_{t-4}{-0.006d}_{t-2}{-0.008d}_{t-4}{-0.005d}_{t-5}{+0.001r}_{t-4}$$

Fig. 3 shows the panel of the number of newly infected, death, and recovery cases, which are compared to the monthly change in the New York stock market. The plots demonstrate a variation in the trend line of each factor. The above figure indicates the New York stock exchange, while the below graph represents pandemic factors, and it is clear that the pandemic began in February 2021 and increased in April. The stock price of New York was 18592 in January 2021, with the highest being 31188 in January 2021, indicating a 67.7% fluctuation in stock prices during this period. Following April, the stock price of New York has no negative influence as compared to the volatility of pandemic instances. The numbers compare the impacts of COVID-19, and all stock exchanges fell with the peak of COVID-19 cases and then rose with the decrease of COVID-19 instances. This demonstrates that the COVID-19 epidemic impacted all stock markets.

**Fig. 4:**
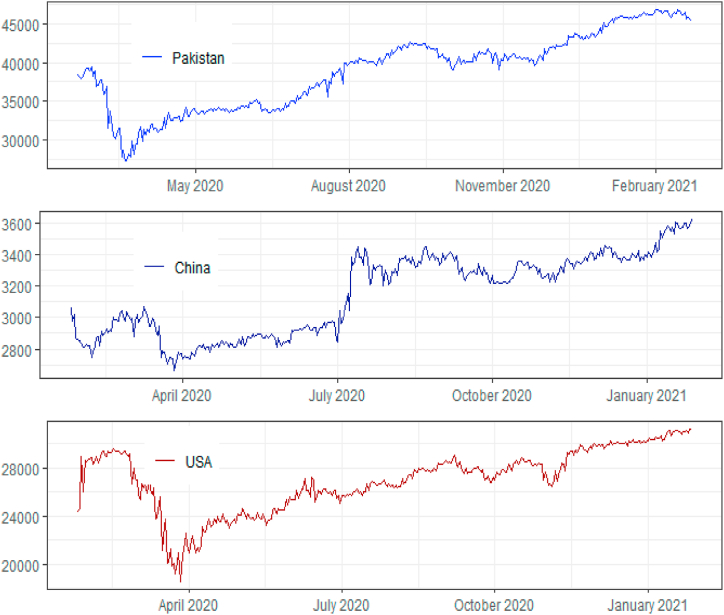
Pakistan, shanghai, and New York stock exchange.

Fig. 4 shows the complete panel of the three stock markets (Pakistan, Shanghai, and New York) had a negative substantial influence at the same time in April because of COVID-19 incidents. Furthermore, there is an increase at various times with distinct fluctuation, we can say about the fluctuation in the three stock markets Pakistan at 72.3%, Shanghai at 36.12%, and New York at 67.7% in the research period owing to COVID-19 instances. Furthermore, the statistics suggest that all nations were impacted, and the number of new confirmed cases grew over time. The United States of America is the most afflicted country, with a high number of confirmed deaths.

**Fig. 5:**
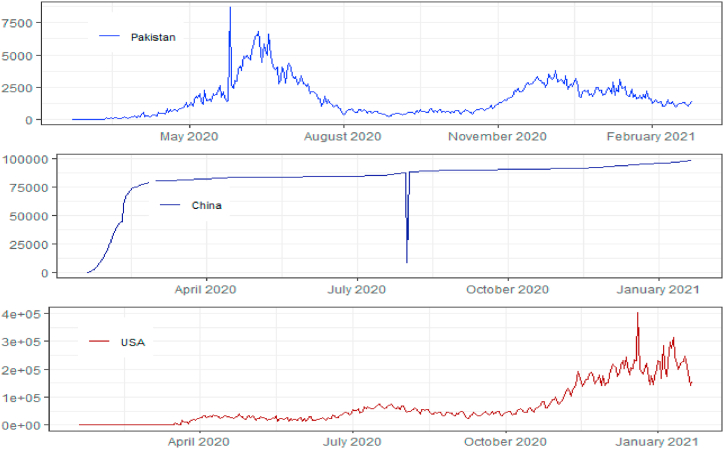
Pakistan, China, and the USA confirm Cases.

In Fig. 5, the panel portrays a one-year trajectory of newly infected COVID-19 cases beginning, rising, and falling, and so on for the three countries Pakistan, the United States (US), and China. China was the first nation where the pandemic began; in August, it dropped to a lower value of 547, and the trend line returned to its original level, remaining constant until January 2021. Following the United States, the number of cases continues to rise and fall from February 2020 to January 2021, reaching a peak point in December. Pakistan is afflicted immediately after China, with the highest number of persons sick owing to COVID-19 in June and July gradually decreasing to October and fluctuating again up to January 2021. We find that Pakistan is doing better than China and the United States in terms of new confirmed cases. There might be a variety of causes for disparities in mortality instances between countries, such as geographic locations, the number of lockdowns, isolation centers, rehabilitation, and so on.

**Fig. 6:**
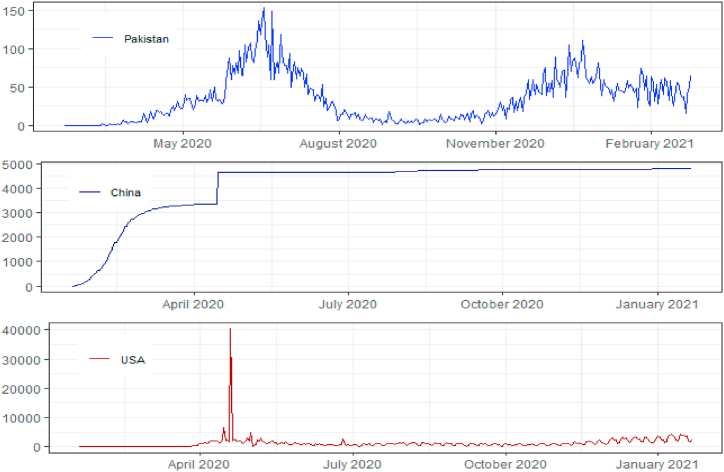
Pakistan, China, and the USA deaths cases.

The complete panel represented by Fig. 6 illustrates a one-year timeline for new COVID-19 mortality cases, showing the rise and decline and so on for three countries: Pakistan, China, and the United States. According to the graph, China had more mortality instances than the other two nations. It reached its peak in April and then remained on the street until the survey ended. The United States experienced its first death case at the end of March, with the largest number reported in April and then drastically decreasing up to the end of time. The death cases in Pakistan with the highest value of 153 then fluctuate further and it is observed that at the same time in the United States and China, death cases pick up in the United States highly and down from China; further China remains up until the finish. In Pakistan, the number of instances fluctuates between the two nations, with a growing and declining trend. The three variables were compared at the same time in the figure to show the variation. The next graph is made up of three panels for recovery scenarios, each of which plays a significant part in the stock market and has a beneficial influence on the stock exchange.

**Fig. 7:**
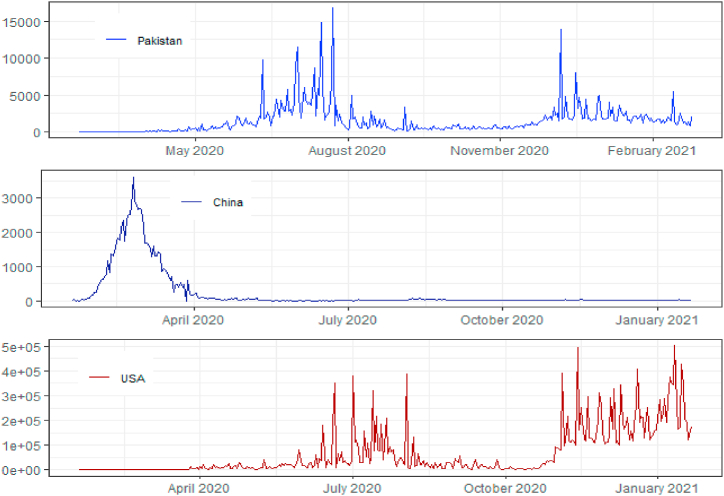
Pakistan, China, and the United States recovery cases.

In Fig. 7, the panel demonstrates the one-year timeframe for COVID-19 recovery cases beginning to grow and decline in three countries: Pakistan, China, and the United States. The following two graphs show that in July 2020, the number of recovery cases in the United States and Pakistan is growing and dropping at the same time. In December, the number of cases increased significantly, but there were fewer of them in Pakistan. And China registered a peak figure of 3622 in March, with no further change.

Table 10 provides an overall overview and comparison highlight, demonstrating variation, Coefficient of Variation, and effect across three stock exchanges: Pakistan (PSX), New York (NYSE), and Shanghai (SSE), as well as COVID-19 examples. Confirm cases had a negative significant impact on the three stock markets, recoveries had a positive significant impact, and death cases had a negative significant impact (NYSE). While volatility and coefficient of variation are at an all-time high in the Pakistan stock market, followed by NYSE, and SSE respectively.

**Table 10:** Overall summary of stock exchanges and COVID-19 cases.

Stock Exchanges	Confirms	Deaths	Recoveries	Fluctuation	C.V
PSX	-ve significant	Insignificant	+ve significant	72.3%	12.44%
NYSE	-ve significant	-ve significant	+ve significant	67.7%	9.7%
SSE	-ve significant	insignificant	+ve significant	36.1%	8.06%

## 2 Conclusion

The significance of COVID-19 on the three stock exchanges in Pakistan, Shanghai, and New York is examined in this research. The Auto-Regressive Distributed Lag (ARDL) model was used in this study, and data were collected from January 2020 to January 2021 to capture the influence of three stock markets. This study aims to determine the relationship between COVID-19, stock exchange fluctuations, and changes in economic policy. According to the ARDL model, the pandemic had a negative influence on three stock markets at the start of COVID-19 instances since the virus transmits very swiftly from person to person daily. Furthermore, the graph shows that the stock market has a negative influence at the start of COVID-19, followed by a gradual upward trend up to the end of the research in the three stock markets. The study also looks at the fluctuation parallels and dissimilarities that develop with a high degree in Pakistan's stock trend 72.3%, USA's 67.7%, and China's 36.1%. We concluded that the confirmed cases harm stock markets, recovery is positive, and the fatalities effect is modest in China and Pakistan but substantial in New York.

## Author contribution statement

All authors listed have significantly contributed to the development and the writing of this article.

## 3 Data availability statement

The data was taken from Pakistan, Shanghai, and New York stock exchanges website whereas the COVID-19 pandemic data was taken from respective countries health department websites.

## Declaration of competing interest

The authors declare that they have no known competing financial interests or personal relationships that could have appeared to influence the work reported in this paper.
